# Lipid and fatty acid metabolism in trypanosomatids

**DOI:** 10.15698/mic2021.11.764

**Published:** 2021-10-06

**Authors:** Giovana Parreira de Aquino, Marco Antonio Mendes Gomes, Roberto Köpke Salinas, Maria Fernanda Laranjeira-Silva

**Affiliations:** 1 Department of Physiology, Institute of Biosciences, University of São Paulo, São Paulo, Brazil.; 2 Department of Biochemistry, Institute of Chemistry, University of São Paulo, São Paulo, Brazil.

**Keywords:** Trypanosoma brucei, Trypanosoma cruzi, Leishmania, neglected tropical diseases, host-parasite interaction, chemotherapeutics, elongases

## Abstract

Trypanosomiases and leishmaniases are neglected tropical diseases that have been spreading to previously non-affected areas in recent years. Identification of new chemotherapeutics is needed as there are no vaccines and the currently available treatment options are highly toxic and often ineffective. The causative agents for these diseases are the protozoan parasites of the Trypanosomatidae family, and they alternate between invertebrate and vertebrate hosts during their life cycles. Hence, these parasites must be able to adapt to different environments and compete with their hosts for several essential compounds, such as amino acids, vitamins, ions, carbohydrates, and lipids. Among these nutrients, lipids and fatty acids (FAs) are essential for parasite survival. Trypanosomatids require massive amounts of FAs, and they can either synthesize FAs *de novo* or scavenge them from the host. Moreover, FAs are the major energy source during specific life cycle stages of *T. brucei, T. cruzi*, and *Leishmania*. Therefore, considering the distinctive features of FAs metabolism in trypanosomatids, these pathways could be exploited for the development of novel antiparasitic drugs. In this review, we highlight specific aspects of lipid and FA metabolism in the protozoan parasites *T. brucei, T. cruzi,* and *Leishmania* spp., as well as the pathways that have been explored for the development of new chemotherapies.

## INTRODUCTION

Trypanosomiases and leishmaniases are vector-borne diseases caused by protozoan parasites of the Trypanosomatidae family. Both are considered neglected tropical diseases as they prevail in tropical and subtropical developing countries, particularly affecting those living in poverty. In addition, currently available treatments are often toxic and ineffective. There are no available vaccines, therefore, effective control can only be achieved by population-based public health interventions [1].

Human African trypanosomiasis (HAT, also known as sleeping sickness) is caused by the hemoflagellate *Trypanosoma brucei*. HAT is endemic in sub-Saharan Africa, affecting mainly rural areas [2]. The disease is transmitted by the injection of metacyclic trypomastigotes into the mammalian host skin through the bite of an infected tsetse fly [2]. The parasites proliferate and later establish bloodstream infection [3]. The bloodstream forms (BSFs) are found in the blood, lymph, and cerebrospinal fluid. American trypanosomiasis, also known as Chagas disease, is caused by *Trypanosoma cruzi*, and is transmitted to humans by contact with feces and/or urine of infected blood-sucking triatomine insects. In the mammalian host, the infective metacyclic trypomastigote forms penetrate the dermis, reach the blood, and infect all types of nucleated cells, where they differentiate into intracellular amastigote forms [4]. Chagas disease is endemic in South and Central Americas but due to environmental changes and migration, it has been spreading to previously non-affected areas including countries in Europe [5], North America [6], Africa, and Western Pacific regions [7]. This disease can also be transmitted through oral non-vectorial routes by ingestion of contaminated food, such as açai pulp products [8, 9] and sugarcane juice [8]. Leishmaniases are caused by at least 22 species of the genus *Leishmania* [10]. The infective metacyclic forms of these parasites are transmitted by hematophagous phlebotomine sandflies during a blood meal. Inside the vertebrate host, *Leishmania* parasites infect phagocytes such as neutrophils and macrophages, where they differentiate into intracellular amastigote forms [10]. These diseases are endemic in 98 countries worldwide, affecting mainly poor regions of South America, Africa, and South-East Asia [11]. Leishmaniases have also been spreading to other regions due to environmental changes, migration, and socioeconomic factors [1, 12].

During their life cycle, trypanosomatid parasites undergo significant environmental changes in temperature, pH, and nutrients availability as they alternate between invertebrate and vertebrate hosts [13, 14]. To survive and thrive, they must adapt to these different environments and compete with their hosts for several essential compounds including amino acids, vitamins, ions, carbohydrates, and lipids [13, 15–18]. Among these nutrients, lipids and fatty acids (FAs) are essential for parasite growth and survival [19, 20].

Trypanosomatids can either synthesize FAs *de novo* or scavenge them from the host. These parasites require substantial amounts of FAs to build the lipid anchors of the glycosylated proteins that decorate the parasite cell surface [21]. For instance, myristate is synthesized intracellularly and esterified to glycosylphosphatidylinositol (GPI) [21], which is a molecule that anchors surface proteins like the variant surface glycoproteins (VSGs), essential for immune system evasion by *T. brucei* BSFs [21]. In addition, disruption of mitochondrial FAs synthesis affects the morphology and function of *T. brucei* mitochondria [20]. It was also observed that lipid extracts of *Leishmania infantum* may induce macrophage polarization into the M2 phenotype [22], promoting pathogen replication rather than elimination [23]. Besides, during specific life cycle stages of *T. brucei, T. cruzi*, and *Leishmania*, FAs are oxidized and become the major energy source [24–26]. Noteworthily, some parasite forms can accumulate in the host adipose tissue [24, 27]. In this niche, scavenging FAs from adipocytes may be advantageous to fulfill the parasite nutritional needs, as observed in *T. brucei* [24].

## LIPID AND FATTY ACID ACQUISITION AND STORAGE

**Figure 1:**
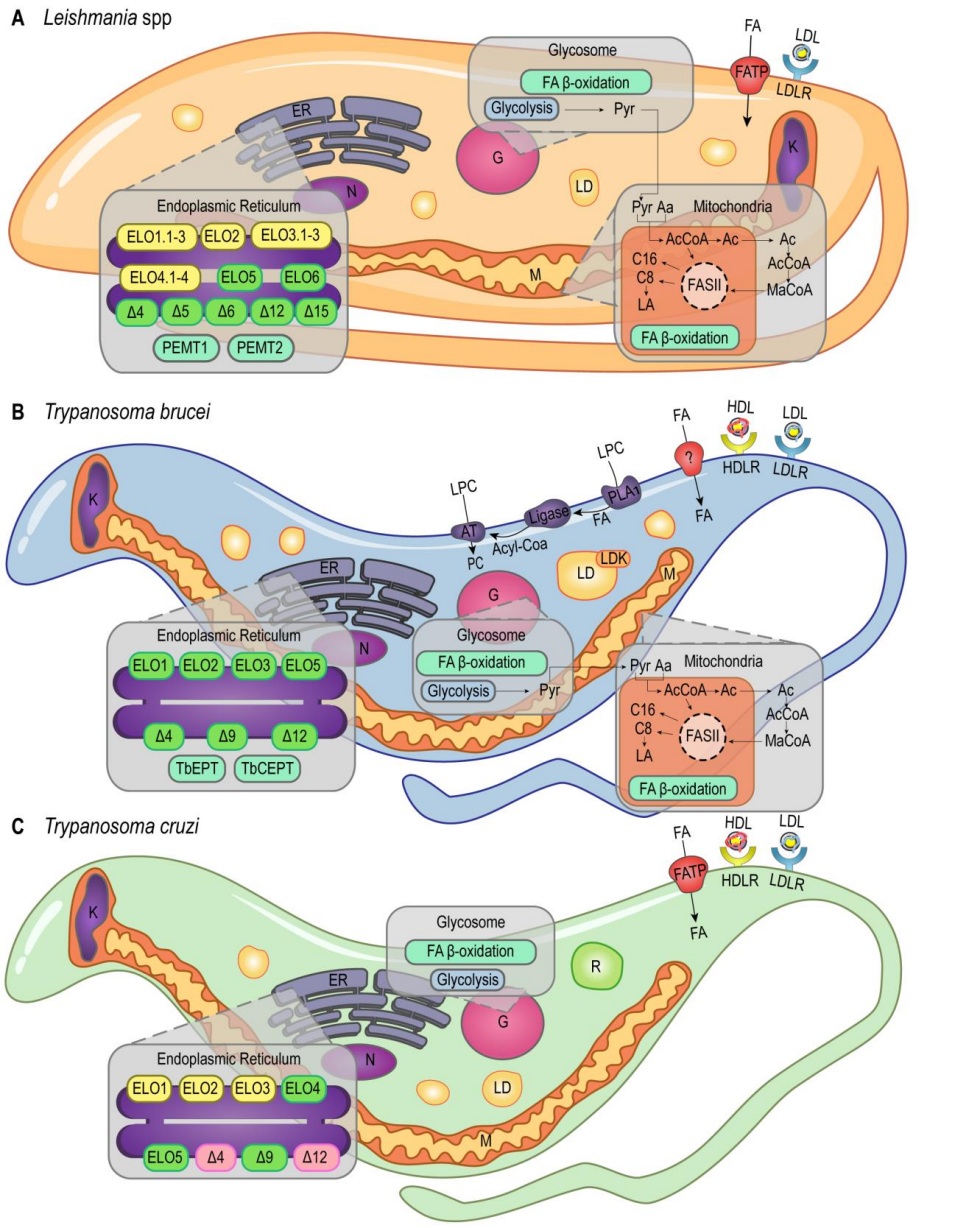
FIGURE 1: Lipid and FA pathways in *Leishmania* spp., *T. brucei* and *T. cruzi*. Schematic illustration of lipid and FA acquisition and metabolic pathways identified in *Leishmania* spp. **(A)**, *T. brucei* **(B)** and *T. cruzi* **(C)**. Aa - Aminoacid; AcCoA - Acetyl CoA; AT - Acetyltransferase; C8 - Octanoic acid; C16 - Palmitic acid; ELO1.1-3, ELO2, ELO3.1-3, ELO4.1-4, ELO5, ELO6 - Elongases; ER - Endoplasmic Reticulum; FA - Fatty Acid; FASII - Fatty Acid Synthase II; FAT - Fatty Acid Transporter; G - Glycosome; HDL - High Density Lipoprotein; HDLR - High Density Lipoprotein Receptor; K - Kinetoplast; LA - Lipoic acid; LD - Lipid Droplets; LDK - Lipid Droplet Kinase; LDL - Low Density Lipoprotein; LDLR - Low Density Lipoprotein Receptor; LPC - Lysophosphatidylcholine; M - Mitochondria; MaCoA - Malonyl-CoA; N - Nucleus; PC - Phosphatidylcholine; PEMT1 - Phosphatidylethanolamine Methyltransferase 1; PEMT2 - Phosphatidylethanolamine Methyltransferase 2; PLA1 - Phospholipase 1; Pyr - Pyruvate; R - Reservosomes; TbCEPT - Choline/Ethanolamine Phosphotransferase; TbEPT - Ethanolamine Pphosphotransferase; Δ4-6, Δ9, Δ12, Δ15 – Desaturases. Yellow boxes, *Leishmania* spp. putative genes (ELO1.1-3, ELO2, ELO3.1-3, ELO4.1-4 in A). Green boxes, characterized proteins (ELO5, ELO6 and desaturases in A; ELO1, ELO2, ELO3, ELO5 and desaturases in B; ELO5 and Δ9 in C). Pink boxes, protein activity detected in *T. cruzi* (Δ4 and Δ12 in C).

The endocytic process is a well-established pathway of FA acquisition that occurs almost exclusively in the flagellar pocket of trypanosomatids (reviewed in [28]; **Figure 1**). As demonstrated by heavy water labeling experiments, *Leishmania mexicana* amastigotes derived from skin lesions appear to be highly dependent on myristic, palmitic, and palmitoleic acid salvage pathways, although it's not yet clear how these molecules are internalized [29].

*T. brucei, T. cruzi,* and *Leishmania amazonensis* must acquire cholesterol from the medium as they are not able to synthesize it. They are capable of internalizing low-density lipoprotein (LDL) by receptor-mediated endocytosis [30–33]. *T. cruzi* and *T. brucei* can also internalize high-density lipoprotein (HDL) through receptor-mediated endocytosis (**Figure 1B**, **1C**) [34–37]. Despite being able to synthesize ergosterol, it was shown that *T. brucei* BSFs meet their sterol requirement via host cholesterol uptake, while procyclic forms (PCFs) rely mainly on *de novo* ergosterol biosynthesis. Importantly, cholesterol uptake seems to suppress C28-sterol synthesis in *T. brucei* BSFs [38]. In addition, *T. brucei* BSFs present an extra three-step pathway to acquire lysophospholipids (LPCs) from the host: hydrolysis mediated by phospholipase A1 (PLA1), esterification with coenzyme A, and acylation mediated by an acyltransferase (AT; **Figure 1B**). The first step of this pathway releases free FAs in the cytoplasm that can also be used in other pathways [39].

Although FA uptake was detected in these parasites, the mechanisms of FA incorporation are not fully understood. Bioinformatic studies indicate the presence of Fatty Acid Binding Protein (FABP)-like proteins in trypanosomatids with a potential role in uptake and transport of FA [37]. These studies showed the *T. brucei* mitochondrial aspartate aminotransferase (AST) presents high amino acid sequence identity to the mammalian plasma membrane FABP (FABPpm), suggesting that *T. brucei* may use this FABPpm-like AST in a similar manner to scavenge FA from the host. Moreover, since FAs concentration in plasma is low, bovine serum albumin (BSA) endocytosis also represents an important route for FA uptake, as shown with *T. cruzi* epimastigotes and *T. brucei* BSFs [28].

Numerous structurally different FABPs have already been identified in other pathogenic organisms such as *Echinococcus granulosus* [40], *Schistosoma mansoni* [41]*, Ascaris suum* [42], *Mycobacterium tuberculosis* [43], *Staphylococcus aureus* [44, 45], and *Streptococcus pneumoniae* [46]. In *S. aureus* and *S. pneumoniae*, FABPs called FakBs mediate incorporation of exogenous FAs [44]. FakB exchanges its bound acyl-phosphate for a membrane FA. Then the glycerol 3-phosphate acyltransferase (PlsY) utilizes the released acyl-phosphate to produce lysophosphatidic acid, which is required for phospholipid (PL) synthesis [44]. X-ray crystallographic analysis of FakB revealed a two-domain protein fold forming a narrow tunnel that accommodates a FA ligand [47, 48]. In contrast, the FABPs of the nematode worms *E. granulosus, S. mansoni, and A. suum* are structurally similar to mammalian FABPs. In mammalian cells, FABPs are also involved in FA trafficking [49] and display a β-barrel structure. This structure encloses a solvent accessible cavity that accommodates the ligand, and a helix-turn-helix motif on one side of the β-barrel covers the cavity [42, 50, 51]. FABP-like proteins displaying the β-barrel fold were also observed in some Gram-positive bacteria. For instance, *M. tuberculosis* studies identified Rv0813c as a hypothetical protein with high similarity to the β-barrel FABPs of mammals. Such findings uncovered a new family of bacterial proteins structurally similar to eukaryotic cellular retinol-binding proteins (CRBPs), cellular retinoic acid-binding proteins (CRABPs), and FABPs [43].

Regarding storage, *T. cruzi* epimastigotes accumulate exogenous lipids in round-shaped genus-specific organelles called reservosomes (**Figure 1C**) [16], the last compartment of the epimastigotes endocytic pathway [28]. The major content of these unique organelles is neutral lipids, mainly cholesterol and cholesteryl esters. It was shown during serum starvation that epimastigotes can mobilize high amounts of lipids from reservosomes to meet cellular demands [16], and the internalized cholesterol is redistributed to the plasma membrane in starved parasites [52]. Furthermore, previous studies have demonstrated that reservosomes undergo a progressive involution during metacyclogenesis, suggesting the storage of lipids in these organelles may be particularly important during parasite differentiation [16, 53].

Lipid droplets (LDs), or lipid bodies (LBs), are other lipid-storage compartments found in the cytosol of *T. brucei* [54], *Leishmania major* intracellular amastigotes [55] and *T. cruzi* epimastigotes [56] (**Figure 1**). These organelles are delimited by a PL monolayer [57] and they accumulate exogenous cholesterol in the form of cholesteryl-esters [58]. However, while reservosomes lipid stocks are preferentially consumed over LDs stocks during lipid starvation in *T. cruzi* epimastigotes [52], LDs could have a role during infection [59]. As demonstrated in transmission electron microscopy (TEM) studies, the production of LDs in the cytoplasm of *T. cruzi* trypomastigotes is a response to host interaction and inflammatory signals. These studies have also shown that exogenous arachidonic acid (AA) stimulates LDs formation that then incorporate AA [60]. Besides, parasite LDs can act as a site for prostaglandin E2 (PGE_2_) production, reinforcing LDs role in disease outcome [60]. Similarly, during *Leishmania infantum chagasi* metacyclogenesis, LDs are intracellular sites of prostaglandin production promoting macrophage infection [61]. It was shown that oleate is incorporated into PLs and triacylglycerols (TAGs) for storage in LDs of *T. brucei* PCFs [62]. RNAi experiments revealed that LDs biogenesis in these parasites relies on a protein kinase, called lipid droplet kinase (LDK) [54], and a lipin-like enzyme that converts phosphatidic acid PLs into diacylglycerol (DAG), called TbLpn, which is essential for PCFs growth [63]. Colocalization assays indicated that LDK is located on the PL monolayer of LDs, whereas TbLpn is distributed throughout the cytosol.

## FATTY ACID BIOSYNTHESIS

Unlike other eukaryotes, trypanosomatids use the elongase (ELO) system to synthesize most of the required FAs [37, 64, 65]. The ELO enzymes are endoplasmic reticulum (ER) integral membrane proteins that catalyze the extension of acyl chains. *T. brucei* uses a modular system of three ELOs to synthesize FAs from C4 to C18, TbELO1-3 (**Figure 1B**) [64]. TbELO1 extends C4 to C10, TbELO2 extends C10 to C14, and TbELO3 extends C14 to C18. Noteworthily, the downregulation of TbELO3 in BSFs may cause the accumulation of myristate (C14), an important component of the VSGs GPI-anchors as discussed earlier [21, 64].

*T. cruzi* and *L. major* genomes also have genes encoding putative ELO1-3 proteins (**Figure 1A**, **1C**), although they were shown to encode additional ELOs [64]. ELO5 synthesizes very long-chain FAs (VLCFAs) in *T. cruzi*, extending C18 to C26. The major product of ELO5 is C24, which is part of the GPI-anchors of infective trypomastigotes [66, 67], while C26 seems to be involved in the synthesis of sphingolipids (SLs) [68]. In *L. major,* ELO5 and ELO6 are involved in the extension of polyunsaturated FAs [69]. Moreover, ELO3.3, ELO4.2, and ELO4.3 expression is upregulated in *L. major* nectomonad promastigotes, indicating that biosynthesis of FAs increases in response to deprivation and stress conditions at this parasite life stage [70]. Noteworthily, the nectomonad promastigotes are the migratory forms found in the sand fly vector that will attach to the midgut epithelium of the insect mediating parasite establishment rather than expulsion, and *L. major* lipophosphoglycan (LPG) is the parasite surface molecule responsible for attachment [71].

Despite the unconventional ELO pathway, *T. brucei* and *Leishmania* spp. also use a mitochondrial type II FA synthesis pathway (FAS II) [72–74]. Unlike the cytosolic FAS I pathway, the mitochondrial FAS II relies on separate polypeptides that mediate the synthesis of caprylate (C8) and palmitate (C16; **Figure 1A**, **1B**) [73]. Caprylate is a precursor of lipoic acid (LA) synthesis, an important cofactor of mitochondrial pyruvate dehydrogenase. In another study, researchers identified three *L. major* genes coding for at least two of the four enzymes involved in the FAS II pathway. Two of these *L. major* genes were shown to encode the 3-hydroxyacyl-ACP dehydratase, but the evolutionary advantages of this gene duplication are not yet clear [74]. In *T. cruzi*, however, no gene related to the mitochondrial FAS II system has been reported.

Genes involved in modification of FAs were also found in trypanosomatids genome databases, including those involved in the cyclopropanation [75, 76] and synthesis of unsaturated FAs, which are FA molecules with one or more carbon-carbon double-bonds [77, 78]. For instance, *Leishmania* spp., except *L. major*, encode a cyclopropane FA synthase (CFAS), whereas *Trypanosoma* lack this gene [76]. In eukaryotes, CFAS is responsible for the addition of a methylene group to carbon-carbon double-bonds of unsaturated FAs in membrane lipids, forming cyclopropane FA (CFA). CFAs synthesis was shown to affect the cellular shape of *L. mexicana* and its resistance to acidic environments, although CFAS knockout does not appear to have any impact on parasite infectivity [75]. In *L. infantum* promastigotes, on the other hand, absence of CFAS significantly decreased activity of membrane transporters and diminished parasite burdens in the spleen and liver during *in vivo* infection [76].

Furthermore, desaturases were identified in *T. brucei* [79]*, L. major*, and *T. cruzi* [77]. These enzymes were shown to be responsible for the synthesis of unsaturated and polyunsaturated FAs by insertion of double bonds at specific sites of the FA carbon skeleton [77]. In *L. major*, Δ4, Δ5, Δ6, Δ12, and Δ15 desaturases were characterized, whilst in *T. brucei* and *T. cruzi*, only Δ4, Δ9, and Δ12 desaturases were identified [77, 78] (**Figure 1**). Deuterium labeling studies revealed that *L. mexicana* amastigotes present high amounts of intracellular oleic (C18:1) and linoleic (C18:2) FAs, which are dependent on the presence of Δ12 desaturase activity [29]. Since, Δ12 desaturase activity is not detected in mammals, this enzyme was proposed as a promising drug target for novel chemotherapeutics against leishmaniases and trypanosomiases [80–82].

Acetyl-CoA is a key metabolite linking many biosynthetic and catabolic pathways, including biosynthesis and degradation of FAs. In particular, trypanosomatids have developed distinct mechanisms to obtain acetyl-CoA. This metabolite is mostly derived from threonine degradation [65, 83, 84], glucose (via pyruvate), or leucine, and it is incorporated into FAs by the mitochondrial FAS II pathway or the ELO pathway in the ER [83] (**Figure 1A** and **1B**). Threonine is salvaged from extracellular sources [85] and it is the preferred carbon source for lipid biosynthesis in *T. brucei* [84, 86]. On the other hand, genes involved in the threonine degradation pathway are absent in *Leishmania* spp. genomes, implying that *Leishmania* may incorporate other precursors to synthesize acetyl-CoA and acetate. For instance, in *L. mexicana* promastigotes, acetate is synthesized from aspartate and acetyl-CoA is produced by the catabolism of ketogenic amino acids [87].

Another difference to other eukaryotes is that the citrate/malate shuttle, which transfers acetyl-CoA from the mitochondrial matrix to the cytosol, is not essential in *T. brucei* PCFs. The inhibition of citrate lyase was shown to have no effect on the incorporation of radioactive-labeled glucose into lipids [85]. Alternatively, *T. brucei* uses a membrane carrier for translocation of acetate from the mitochondria to the cytosol, where acetate is converted back to acetyl-CoA by a cytosolic acetyl-CoA synthetase (ACS) at the expense of ATP. In mitochondria, acetate is synthesized from acetyl-CoA by the action of two mitochondrial enzymes, acetyl-CoA thioesterase (ACH) and acetate:succinate CoA-transferase (ASCT), which is involved in substrate-level ATP production together with succinyl-CoA synthetase [88–91]. In the cytosol, acetyl-CoA is carboxylated to malonyl-CoA by acetyl-CoA carboxylase (ACC). Yet, the origin of malonyl-CoA required in the mitochondrion is unclear [92]. In *T. brucei*, ACC was shown to be required for elongation of FAs in both parasite life stages. In PCFs, ACC activity and protein levels were downregulated under lipid-rich growth conditions. However, this was not observed in BSFs, most likely because these forms require constitutively active ACC for myristate synthesis and GPI-anchor assembly [93].

## LIPID BIOSYNTHESIS

Upon synthesis, FAs can be incorporated into more complex molecules, such as PLs which are the major components of biological membranes in eukaryotes. Numerous studies on lipid composition of PCFs and BSFs of *T. brucei* have shown that phosphatidylethanolamine (PE) and phosphatidylcholine (PC) are the major classes of PLs in *T. brucei* (reviewed in [94]). A similar lipid profile was also observed in trypomastigotes and amastigotes of *T. cruzi*, and amastigotes and promastigotes of *Leishmania donovani* and *L. infantum* [95, 96]. PE and PC are synthesized via the two branches of the Kennedy pathway (reviewed in [97]) that consists of three conserved enzymatic reactions. The first step of this pathway is catalyzed by a kinase, which promotes the phosphorylation of ethanolamine and choline. The second step is catalyzed by a cytidylyltransferase, forming the high energy intermediates CDP-ethanolamine and CDP-choline. And the third step is catalyzed by a phosphotransferase that transfers the ethanolamine and choline intermediates to DAG and alkyl-acylglycerol (AAG) [97]. The kinases of both branches of the Kennedy pathway were identified and biochemically characterized in *T. brucei* [98], as well as the cytidylyltransferase of the ethanolamine branch, shown to be essential in *T. brucei* BSFs [99]. Moreover, the phosphotransferase from the choline branch was localized to the ER, and the phosphotransferase from the ethanolamine branch was localized to a sub-compartment close to the nuclear membrane [100]. In *L. infantum*, the kinase from the choline branch was also shown to phosphorylate ethanolamine, albeit with much lower efficiency compared to choline [101]. Besides, two PE methyltransferases, which convert PE into PC through tri-methylation, were identified in the ER of *L. major*. Noteworthily, both enzymes are inhibited by the choline analogs miltefosine and hexadecyltrimethylammonium bromide, supporting their potential as drug targets [102]. Furthermore, it was shown that PC synthesis is essential for *L. major* promastigotes, but not for amastigotes [103]. To date, no components of the Kennedy pathway have been characterized in *T. cruzi*.

SLs are other major lipid components of trypanosomatids biological membranes. Genomic database searches identified most of the genes involved in the sphingolipid biosynthetic pathway in trypanosomatids [104]. The enzymes of the final step of this pathway, sphingolipid synthases (TbSLS1-4), were characterized in *T. brucei* [105]: TbSLS1 produces inositol phosphorylceramide (IPC), TbSLS2 produces ethanolamine phosphorylceramide (EPC), and both TbSLS3 and TbSLS4 produce sphingomyelin (SM) and EPC. Importantly, IPC and EPC are rare lipid species in mammalian hosts [106], hence, their synthesis pathways are potential targets for drug development. Furthermore, in *L. major*, the SL metabolic pathway can be redirected for production of the ethanolamine required for synthesis of PE by the Kennedy pathway [107] (for a more detailed review on SL and PL metabolism in *Leishmania* see [108]).

Sterols are other important membrane components that play a crucial role in regulating membrane dynamics and fluidity. Metabolomic analyses revealed ergosterol as the major cellular sterol in *Leishmania* [109]. Genome database searches identified putative genes of the isoprenoid pathway potentially involved in the synthesis of ergosterol in *T. brucei, T. cruzi,* and *L. major* [110]. Proteomic analysis of purified glycosomes from *T. cruzi* epimastigotes revealed the compartmentalization of several enzymes associated with the isoprenoid pathway, also referred as the mevalonate pathway [111]. Other studies also located the enzymes of the early steps of isoprenoid pathway in the glycosomes of *T. brucei* and *L. major* [112]. Compartmentalization of the isoprenoid pathway may have a role on the modulation of sterol synthesis throughout parasite differentiation, as observed for other pathways [113]. Accordingly, significant differences in sterol composition were observed between *L. infantum* procyclic and metacyclic promastigotes [114].

## FATTY ACID OXIDATION

FAs can also be oxidized to provide energy through the β-oxidation pathway that may occur in the mitochondria or in the glycosomes of trypanosomatids (**Figure 1**) [110, 111, 115, 116]. Glycosomes are unique trypanosomatid organelles related to eukaryotes' peroxisomes. Uptake of long-chain fatty acyl-CoA esters into peroxisomes was shown to be mediated by the peroxisomal ATP-binding cassette protein subfamily D (ABCD) in plants, and by the peroxisomal long-chain fatty acid import proteins 1 and 2 (PXA1 and PXA2) in yeast [117–119]. Similarly, glycosomal ABC transporters 1 to 3 (GAT 1-3), homologs of the peroxisomal ABC transporters, were identified in the glycosomal membrane of *T. brucei* [120, 121]. The knockdown of GAT1 by RNA interference (RNAi) in PCFs of *T. brucei* showed a significant increase of linoleate (C18:2) and a decrease of glycosomal oleoyl (C18:1)-CoA incorporation, supporting GAT1 role in FAs transport from the cytosol into the glycosome [122].

Noteworthily, stage-specific changes in carbon metabolism were observed in *Leishmania*. These studies revealed that *L. mexicana* intracellular amastigotes exhibit reduced glucose consumption compared to promastigotes, which was compensated by increased β-oxidation of FAs [113]. In addition, enzymes involved in the β-oxidation of FAs were shown to be upregulated in *L. donovani* and *L. major* amastigotes [123]. On the other hand, trypanosomes exhibit reduced or undetectable oxidation of FAs during their life cycles. Yet, *T. brucei* forms found in the adipose tissue have a distinct metabolic profile with upregulation of the β-oxidation pathway [24]. *T. cruzi* can also be found in the adipose tissue [124], often near lipid droplets, suggesting that they may also be exploiting local lipolysis to match their energy requirements [27]. Clinically, the findings revealing the adipose tissue as a novel niche for trypanosomes may help understand some cases of relapse, since this tissue is known for its low perfusion for drugs [125, 126].

## FATTY ACID REMODELING

GPI-anchors are lipid-conjugated molecules present in all eukaryotes yet appear in a higher frequency in protozoa [127]. The main role of GPI-anchors is to attach enzymes, receptors, and other macromolecules to the plasma membrane [127]. In protozoan parasites, most GPI-anchors share a common backbone structure composed of EtN-*P*-6Manα1-2Manα1-6Manα1-4GlcNα1-6myo-inositol-1-P lipid, with additional mannosylation or phosphorylation [127, 128]. Within this core structure, several lipid configurations and modifications can be found depending on the parasite species and/or the life stage. Such modifications include the inositol 2-O-acylation in *T. brucei* PCFs, the presence of a chain branch at the C3 position of Mannose I in *T. brucei* VSG GPI-anchors, and the hybrid type Glycoinositol Phospholipids (GIPLs) in *Leishmania* [128]. Besides, GPI-anchors can vary in PL composition, length, and degree of saturation in the hydrocarbon chains [128]. Unlike mammalian GPI-anchors, trypanosomatids GPI-anchors display typical FA acid remodeling. For example, *T. brucei, T. congolense*, and *T. equiperdum* GPI-anchors are exclusively di-myristoylated, while *Leishmania* GP63 displays mono-myristoylated GPI-anchors (reviewed in [19]).

In trypanosomes and *Leishmania*, GPI-anchored glycoconjugates extensively coat the plasma membrane and are involved in several aspects of the host-parasite interaction. Such aspects include survival, host immune response evasion, adhesion and invasion of host cells, and pathogenesis [127]. In addition, the GPI-anchored procyclin proteins may protect *T. brucei* against proteolytic attack in the tsetse midgut [129]. About ten million copies of VSGs were found to cover each BSF of *T. brucei*, and these VSGs are attached to the lipid bilayer of the parasite plasma membrane via a GPI-anchor containing exclusively two myristate molecules [73, 130]. *T. brucei* GPI-VSGs were shown to induce expression of pro-inflammatory genes, such as TNF-α, IL-6, and GM-CSF [131]. *T. cruzi* GPI-anchors were also found to be potent proinflammatory molecules as they trigger the production of nitric oxide, IL–12 and TNF–α by macrophages [66, 132]. In the case of *L. mexicana*, GIPLs biosynthesis requires FA remodeling. Similar to what was shown in *T. brucei*, this FA remodeling reactions are CoA and ATP-dependent, and occur on pre-existing but not on *de novo* synthesized GIPLs [133].

## LIPID AND FATTY ACID METABOLISMS AS DRUG TARGETS

There are few currently available drugs to treat leishmaniases and trypanosomiases [7, 134, 135]. Among the registered anti-*Leishmania* drugs, Miltefosine, Amphotericin B, and pentavalent antimonials (Sb^V^) target lipid-related pathways (**Table 1**). Miltefosine, a PC analog, was originally developed as an antitumoral drug (reviewed in [136]) and is the only oral anti-*Leishmania* drug approved by the United States Food and Drug Administration (U.S. FDA). Although its specific mechanism of action is still unclear, PL quantification of *L. donovani* promastigotes treated with Miltefosine showed a reduction of parasites PC levels and an increase of PE levels [137], suggesting that Miltefosine may interfere with the PE methyltransferase that converts PE into PC. Miltefosine also impairs mitochondrial function and disturbes calcium homeostasis in *L. donovani* [138–140]. Moreover, Miltefosine-treated parasites showed higher amounts of cholesterol than non-treated parasites [137], suggesting that Miltefosine may also promote cholesterol uptake. Amphotericin B deoxycholate (AmB) targets ergosterol precursors leading to disruption of *Leishmania* membrane [141, 142], and it is the preferential treatment for patients co-infected with HIV and visceral leishmaniasis [143, 144]. Its lipid formulation, Liposomal Amphotericin B (AmBisome^®^; LAmB) is a less-toxic alternative [143, 145]. This formulation corresponds to AmB solubilized in unilamellar vesicles composed of cholesterol and PLs, which leads to increased and longer-lasting efficacy [143]. Pentavalent antimonials (Sb^V^) are another group of compounds widely used to treat leishmaniases and appear to disturb FAs synthesis [146]. However, their mechanism of action is also still unclear. In particular, among the Sb^V^, sodium stibogluconate was proposed to inhibit glucose catabolism and FAs β-oxidation in *Leishmania* [147]. Other metabolic studies revealed major differences in lipid metabolism of antimonial-resistant parasites, indicating that these pathways may be related to parasite drug resistance [148].

**Table 1.** Registered drugs and potential inhibitors targeting trypanosomatids' FA and lipid-related pathways.

**Target pathway**	**Inhibitor/Drug**	**Organism**
Ergosterol metabolism	Amphotericin B deoxycholate^*a*^ Liposomal Amphotericin B^*a*^	*Leishmania* [142, 143, 145]
Ergosterol synthesis	26-Fluorolanosterol (26-FL)	*T. brucei* [161]
FA β-oxidation	Sodium stibogluconate^*a*^	*Leishmania* [147]
FA synthesis	Thiolactomycin	*T. brucei, T. cruzi* and *L. donovani* [150]
PL synthesis	Miltefosine^*a*^	*Leishmania* [136]
Sterol metabolism	Risedronate	*T. cruzi* [160]
	Imipramine	*Leishmania* [137, 159]
SL metabolism	Myriocin	*L. braziliensis* [157]
	Tamoxifen	*L. amazonensis* and *L. major* [158]
	3-(oxazolo[4,5-b]pyridine-2-yl)anilide (OXPA)	*T. brucei* [156]
Δ9 desaturase	10-thiastearic acid	*T. cruzi* [82]
	Isoxyl	*T. cruzi* [82]
	GS-456332	*T. brucei* and *T. cruzi* [81]
Δ12 desaturase	Stearolic acid	*T. brucei* and *T. cruzi* [81]
	12- and 13-thiastearic acid	*T. brucei* [155] and *T. cruzi* [82]

a - Registered for clinical use.

PL – phospholipid; FA – fatty acid; SL – sphingolipid.

Efforts are being made to search for other molecules targeting FA and lipid metabolism that could potentially arise as better alternative drugs against leishmaniases and trypanosomiases (**Table 1**). For instance, *in vitro* treatment of *T. cruzi* with ketoconazole reduced the activity of Δ9 desaturase by 90% [149]. Thiolactomycin, an inhibitor of the condensation step of bacterial FAS II that has also been tested as a potential antimalarial drug [150–152], inhibited *T. brucei* FAs synthesis and parasite growth in culture [153]. Thiolactomycin analogues were also able to inhibit growth of *T. brucei, T. cruzi,* and *L. donovani* [150]. Other studies with *T. cruzi* epimastigotes showed that thiastearic acid (TS) isomers inhibit Δ9 and Δ12 desaturases. Δ9 desaturase was inhibited by 10-TS, while Δ12 was inhibited by both 12- and 13-TS [82]. In addition, isoxyl, a thiourea derivative that inhibits *M. tuberculosis* Δ9 desaturase [154], reduced the percentage of unsaturated FAs in *T. cruzi* epimastigotes [82]. In *T. brucei,*12- and 13-TS also inhibited *in vitro* growth of PCFs and BSFs [155]. Moreover, stearolic acid effectively inhibited Δ12 desaturases of *T. cruzi* epimastigotes and *T. brucei* BSFs, and GS-456332, a known inhibitor of human Δ9 desaturase, inhibited *in vitro* growth of both parasites. The combination of these FAs desaturases inhibitors showed a synergistic effect on trypanosomes, with decreased EC50 values [81].

SL metabolism is another potential drug target for development of anti-trypanosomatids therapy. In particular, metabolomic studies indicate that ceramides, central components of SLs, accumulate in BSFs of *T. brucei* treated with the anti-trypanosomal compound 3-(oxazolo[4,5-b]pyridine-2-yl)anilide (OXPA) [156]. In *Leishmania braziliensis* promastigotes, it was shown that myriocin reduces synthesis of IPC, which is the major SL expressed in this parasite developmental form. Myriocin inhibited *Leishmania* growth by 52% and caused morphological alterations [157]. In *L. amazonensis*, IPC and phosphatidylinositol (PI) levels were reduced by the anticancer drug tamoxifen. Tamoxifen also inhibited *L. major* IPC synthase in *in vitro* assays [158]. Imipramine, a common antidepressant, also exhibited antileishmanial activity through inhibition of sterol biosynthesis. Noteworthily, imipramine combined with miconazole, a known inhibitor of ergosterol synthesis in fungi, had an additive effect improving their leishmanicidal activity [159]. Risedronate is another potential drug that targets *T. cruzi* sterol metabolism, affecting parasites growth and the structure of mitochondria and reservosomes [160]. Lastly, *T. brucei* ergosterol biosynthesis was inhibited by 26-Fluorolanosterol (26-FL) impairing PCFs and BSFs *in vitro* growth [161]. Considering their potential to target FA and lipid metabolism pathways *in vitro*, further *in vivo* studies with all these inhibitors will indicate their prospects for clinical use and may unveil better chemotherapeutics against trypanosomatids.

## SUMMARY AND PERSPECTIVES

Lipids and FAs are among the nutrients that trypanosomatid parasites rely on their hosts to survive and replicate. PLs, SLs and sterols play important roles in membrane biology of trypanosomatids. The synthesis of PLs species is essential, and it can also be redirected to SL synthesis. FAs are the building blocks of PLs, one of the major components of eukaryotes' cell membranes. The degree of unsaturation and the presence of cyclopropanation in the FAs acyl chains provide mechanisms to adjust membrane fluidity. Besides, FAs are components of membrane anchors for a large diversity of surface glycoproteins in trypanosomatids, which have essential roles in host-parasite interaction. Long-chain modified FAs, such as prostaglandins stored in LDs, surface glycoproteins, and phosphoglycans also play important roles in host-parasite interaction and immune response. Furthermore, FAs are part of these parasites strategies to adapt to the different environments during their life cycles, serving as an energy source in specific life stages. Despite all the central roles played by FAs on trypanosomatids biology, the parasites mechanisms to scavenge FAs from the host are still poorly understood. Also, there is a knowledge gap regarding FA and lipid metabolic pathways between *T. cruzi, T. brucei*, and *Leishmania* spp. Nevertheless, these pathways have been explored for the design of new anti-trypanosomatids drugs. Several desaturase inhibitors were identified in recent years, however, most of them have not yet been tested *in vivo*. Therefore, elucidating the peculiarities of lipid and FA metabolism of these parasites will continue to be a promising route for the development of novel therapeutic strategies against trypanosomiases and leishmaniases.

## Abbreviations:

AA: – arachidonic acid;
ACC: – acetyl-CoA-carboxylase;
AmB: – amphotericin B;
AST: – aspartate aminotransferase;
BSF: – bloodstream form;
CFA: – cyclopropane FA;
CFAS: – CFA synthase;
DAG: – diacylglycerol;
EPC: – ethanolamine phosphorylceramide;
ELO: – elongase;
ER: – endoplasmic reticulum;
FA: – fatty acid;
FABP: – fatty acid binding protein;
FAS: – FA synthesis;
GPI: – glycosylphosphatidylinositol;
HAT: – human African trypanosomiasis;
IPC: – inositol phosphorylceramide;
LD: – lipid droplet;
LDK: – LD kinase;
PC: – phosphatidylcholine;
PCF: – procyclic form;
PE: – phosphatidylethanolamine;
PL: – phospholipid;
*Sb*^*v*^: – pentavalent antimonials;
SL: – sphingolipid;
VSG: – variant surface glycoprotein.
